# Relation between Birth Weight and Intraoperative Hemorrhage during Cesarean Section in Pregnancy with Placenta Previa

**DOI:** 10.1371/journal.pone.0167332

**Published:** 2016-11-30

**Authors:** Hiroaki Soyama, Morikazu Miyamoto, Hiroki Ishibashi, Masashi Takano, Hidenori Sasa, Kenichi Furuya

**Affiliations:** Department of Obstetrics and Gynecology, National Defense Medical College, Tokorozawa, Japan; Stellenbosch University, SOUTH AFRICA

## Abstract

**Background:**

Placenta previa, one of the most severe obstetric complications, carries an increased risk of intraoperative massive hemorrhage. Several risk factors for intraoperative hemorrhage have been identified to date. However, the correlation between birth weight and intraoperative hemorrhage has not been investigated. Here we estimate the correlation between birth weight and the occurrence of intraoperative massive hemorrhage in placenta previa.

**Materials and Methods:**

We included all 256 singleton pregnancies delivered via cesarean section at our hospital because of placenta previa between 2003 and 2015. We calculated not only measured birth weights but also standard deviation values according to the Japanese standard growth curve to adjust for differences in gestational age. We assessed the correlation between birth weight and the occurrence of intraoperative massive hemorrhage (>1500 mL blood loss). Receiver operating characteristic curves were constructed to determine the cutoff value of intraoperative massive hemorrhage.

**Results:**

Of 256 pregnant women with placenta previa, 96 (38%) developed intraoperative massive hemorrhage. Receiver-operating characteristic curves revealed that the area under the curve of the combination variables between the standard deviation of birth weight and intraoperative massive hemorrhage was 0.71. The cutoff value with a sensitivity of 81.3% and specificity of 55.6% was −0.33 standard deviation. The multivariate analysis revealed that a standard deviation of >−0.33 (odds ratio, 5.88; 95% confidence interval, 3.04–12.00), need for hemostatic procedures (odds ratio, 3.31; 95% confidence interval, 1.79–6.25), and placental adhesion (odds ratio, 12.68; 95% confidence interval, 2.85–92.13) were independent risk of intraoperative massive hemorrhage.

**Conclusion:**

In patients with placenta previa, a birth weight >−0.33 standard deviation was a significant risk indicator of massive hemorrhage during cesarean section. Based on this result, further studies are required to investigate whether fetal weight estimated by ultrasonography can predict hemorrhage during cesarean section in patients with placental previa.

## Introduction

The prevalence of placenta previa is approximately four cases in 1000 pregnancies [1]. Placenta previa is defined as an abnormal implantation of the placenta over or very near the internal cervical os [2]. For pregnant women with placenta previa, cesarean section is recommended as the mode of delivery [1]. Placenta previa is a well-known cause of massive intrapartum hemorrhage that is associated with high mortality and morbidity for both the mother and the neonate [3,4]. Therefore, for these patients, preparation for possible severe perinatal bleeding is necessary and reliable predictive factors of hemorrhage are important [5,6].

Several risk factors of intraoperative massive hemorrhage in patients with placenta previa have been reported [7–14]. Among these factors, those concerning maternal background were high maternal age and previous cesarean section [7,8]. In addition, variables based on sonographic findings were placenta with sponge-like consistency [7], placental adhesion [8], complete placenta previa [9], anterior placentation [8,10], <3-cm cervical length [11], and thick placental edge [12,13]. However, the correlation between birth weight and intrapartum hemorrhage has not been investigated.

The aim of this study was to investigate whether birth weight could reflect massive hemorrhage during cesarean section for pregnant women with placenta previa.

## Materials and Methods

All patients with singleton pregnancies who underwent cesarean delivery due to placenta previa at our hospital between 2003 and 2015 were identified. The maternal histories, sonographic findings, and intraoperative information were extracted from medical and operative records.

We classified placenta previa into major previa and minor previa. If the placenta covered the internal cervical os, it was defined as major previa. If the leading edge of the placenta was in the lower uterine segment but did not cover the cervical os, it was defined as minor previa [14]. The method used to definitively diagnose placenta previa was transvaginal sonography by experienced obstetricians at around 32 weeks of gestation. The patients with placenta previa were followed up by transabdominal sonographic examination at 1-week intervals after 32 weeks of gestation at our institution. For cases of complications with threatened preterm labor, tocolytic agents were used. At our institution, elective cesarean section was performed at 36–37 gestational weeks because the Guideline for Obstetrical Practice in Japan recommends cesarean section for placenta previa until the end of 37 weeks of gestation [15]. However, if persistent warning bleeding with >100 mL blood loss or uncontrollable uterine contractions occurred, an emergency cesarean section was performed. The cesarean section was conducted by at least two experienced obstetricians and one resident physician. During the cesarean section, an ultrasound-guided incision was created for patients in whom the placenta adhered to the anterior uterine wall. For the other patients, cesarean section was performed by making a transverse incision of the uterus at the lower uterine segment. Intraoperative blood loss > 1500 mL including the amniotic fluid during cesarean section was defined as massive hemorrhage according to the definition of the amount of intraoperative bleeding measured from the time of the skin incision to the time of scar closure based on suction count and towel weight. If the blood loss was increased, hemostatic procedures (e.g., gauze tamponade, tamponade balloon, brace sutures) were performed at the surgeon’s discretion. In this study, a definitive diagnosis of adhesive placenta was made at surgery. A myoma-related complication was defined when the myoma was >5 cm because the presence of myoma is a reported risk factor of bleeding during cesarean section [16].

In the present study, we measured the birth weight and calculated the standard deviation (SD) of the birth weight, adjusting for the Japanese Society of Ultrasound in Medicine’s standard growth curve of the respective gestational weeks [17].

Statistical analysis was performed using JMP 10.0.0 software (SAS Institute, Inc., Tokyo, Japan). The cutoff SD value for birth weight was determined by performing an analysis of the receiver operating characteristic (ROC) curve. The *χ*^2^ test and Mann-Whitney *U* test were used to evaluate differences in characteristics. Univariate and multivariate analyses were performed using logistic regression. Statistical significance was defined as a p value < 0.05.

The present study was approved by the Institutional Review Board of National Defense Medical College (confirmation no.: 2409). Informed consent was not obtained, because this study was a retrospective analysis. However, we provided an opportunity to refuse permission to use the data via our college’s website. Records/information of all patients were anonymized and de-identified prior to analysis by one of the member MM. All authors were involved in medical treatment.

## Results

During the study period, 6629 live neonates were delivered at our hospital; of them, 256 (3.9%) singleton pregnancies with placenta previa were identified. The characteristics of the cases are presented in Table 1. The mean birth weight was 2561 g (range, 1114–3820 g). The mean intraoperative hemorrhage amount was 1393 mL (265–6223 mL), and there were 96 cases (38%) of massive hemorrhage.

**Table 1:** Patient characteristics.

Factors		n = 256	
**Maternal age (years)**	**≥35**	113	(44%)
	**<35**	143	(56%)
**Gestational age (weeks)**	**≥37**	149	(58%)
	**<37**	107	(42%)
**Parity**	**Primipara**	126	(49%)
	**Multipara**	130	(51%)
**IVF pregnancy**	**Yes**	20	(8%)
	**No**	236	(92%)
**Repeat cesarean section**	**Yes**	33	(13%)
	**No**	223	(87%)
**Tocolytic agent use**	**Yes**	101	(39%)
	**No**	155	(61%)
**Warning bleeding**	**Yes**	84	(33%)
	**No**	172	(67%)
**Cesarean section mode**	**Emergency**	70	(27%)
	**Elective**	186	(73%)
**Myoma-related complication**	**Yes**	10	(4%)
	**No**	246	(96%)
**Previa classification**	**Major previa**	133	(52%)
	**Minor previa**	123	(48%)
**Main placenta location**	**Anterior wall**	39	(15%)
	**Posterior wall**	217	(85%)
**Uterine incision type**	**Transverse**	241	(94%)
	**Classical**	15	(6%)
**Placental adhesion**	**Yes**	24	(9%)
	**No**	232	(91%)
**Hemostatic procedures**	**Yes**	103	(40%)
	**No**	153	(60%)
**Birth weight (g)**	**mean (range)**	2561 (1114–3820)	
	**≥34 weeks**	2631 (1410–3820)	
	**<34 weeks**	1706 (1114–2364)	
**SD of birth weight**	**mean (range)**	−0.11 (−3.51 to 3.19)	
	**≥34 weeks**	−0.08 (−3.51 to 3.19)	
	**<34 weeks**	−0.29 (−0.266 to 1.76)	
**Intraoperative hemorrhage (mL)**	**≥1500**	96	(38%)
	**<1500**	160	(62%)
	**(mean)**	1393 (265–6223)	

IVF, in vitro fertilization; SD, standard deviation

**Fig 1:**
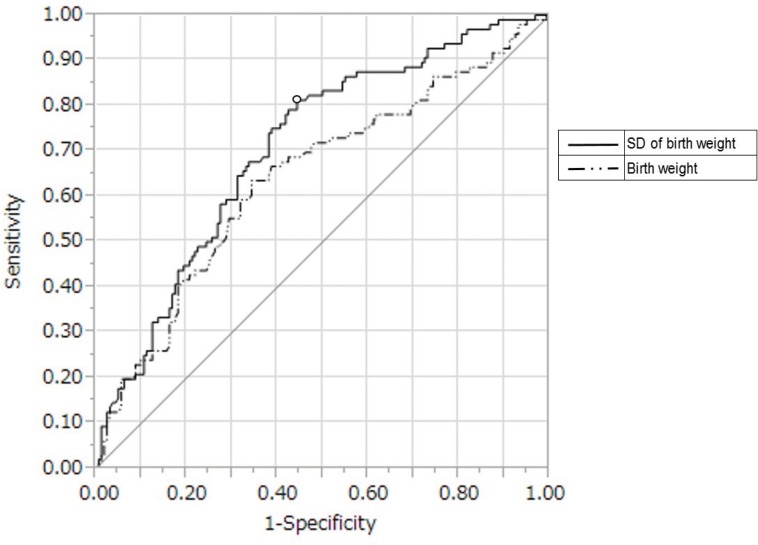
Receiver operating characteristic curve analysis of birth weight for massive intraoperative hemorrhage (≧1500 ml). Areas under the curve of the combination between massive hemorrhage and SD of birth weight or measured value of birth weight are 0.71 and 0.64, respectively. Abbreviation: SD = standard deviation

The ROC curves of the correlations between massive hemorrhage and either the SD or measured birth weights are shown in Fig 1. The area under the curve (AUC) of the combination of massive hemorrhage and SD values (0.71) was higher than the measured values (0.659). In the data set with a cutoff value of −0.33 SD, the sensitivity and specificity were 81.3% and 55.6%, respectively. The characteristics of the >−0.33 SD and <−0.33 SD groups are shown in Table 2. The >−0.33 SD group included 148 cases (58%), all of which had greater blood loss than those in the <−0.33 SD group. No significant differences were found in other factors such as gestational age, parity, in vitro fertilization pregnancy, repeated cesarean section, tocolytic agent use, warning bleeding, mode of cesarean section, myoma-related complications, previa classification, location of main placenta, uterine incision, placental adhesion, or need for hemostatic procedures. Univariate analysis revealed that placental adhesion (odds ratio [OR], 4.70; 95% confidence interval [CI], 1.94–12.60), need for hemostatic procedures (OR, 3.31; 95% CI, 1.86–5.34), and >−0.33 SD of the mean birth weight (OR, 5.08; 95% CI, 2.86–9.37) were related to massive hemorrhage. Multivariate analysis revealed that birth weight >−0.33 SD (OR, 5.88; 95% CI, 3.04–12.00), need for hemostatic procedures (OR, 3.31; 95% CI, 1.79–6.25), and placental adhesion (OR, 12.68; 95% CI, 2.85–92.13) were independent predictors of massive hemorrhage (Table 3).

**Table 2:** Patient characteristics by birth weight (cutoff value, −0.33 SD).

Factors		Birth weight≥−0.33 SD		Birth weight<−0.33 SD		P
		(n = 148)		(n = 108)		
**Maternal age (years)**	**≥35**	69	(47%)	44	(41%)	0.35
	**<35**	79	(53%)	64	(59%)	
**Gestational age (weeks)**	**≥37**	89	(60%)	60	(56%)	0.46
	**<37**	59	(40%)	48	(44%)	
**Parity**	**Primipara**	69	(47%)	57	(53%)	0.33
	**Multipara**	79	(53%)	51	(47%)	
**IVF pregnancy**	**Yes**	14	(9%)	6	(6%)	0.24
	**No**	134	(91%)	102	(94%)	
**Repeat cesarean section**	**Yes**	19	(13%)	14	(13%)	0.98
	**No**	129	(87%)	94	(87%)	
**Tocolytic agent**	**Yes**	64	(43%)	37	(34%)	0.14
	**No**	84	(57%)	71	(66%)	
**Warning bleeding**	**Yes**	51	(34%)	33	(31%)	0.51
	**No**	97	(66%)	75	(69%)	
**Cesarean section mode**	**Emergency**	42	(28%)	28	(26%)	0.66
	**Elective**	106	(72%)	80	(74%)	
**Myoma-related complication**	**Yes**	5	(3%)	5	(5%)	0.61
	**No**	143	(97%)	103	(95%)	
**Previa classification**	**Major previa**	74	(50%)	59	(55%)	0.46
	**Minor previa**	74	(50%)	49	(45%)	
**Main placenta location**	**Anterior wall**	27	(11%)	12	(11%)	0.11
	**Posterior wall**	121	(89%)	96	(89%)	
**Uterine incision type**	**Transverse**	140	(95%)	101	(94%)	0.72
	**Classical**	8	(5%)	7	(6%)	
**Placental adhesion**	**Yes**	15	(10%)	9	(8%)	0.62
	**No**	134	(90%)	99	(92%)	
**Hemostatic procedure needed**	**Yes**	63	(43%)	40	(37%)	0.37
	**No**	85	(57%)	68	(63%)	
**Blood loss (mL)**	**≥1500**	77	(52%)	19	(18%)	<0.0001
	**<1500**	71	(48%)	89	(82%)	

IVF, in vitro fertilization; SD, standard deviation

**Table 3:** Analysis of potential factors contributing to intraoperative hemorrhage.

Factors		*Univariate*			*Multivariate*		
		OR	(95% CI)	P	OR	(95% CI)	P
**Maternal age**	**≥35 years vs. <35 years**	1.11	(0.67–1.86)	0.67	1.26	(0.68–2.34)	0.46
**Gestational age**	**≥37 weeks vs. <37 weeks**	0.82	(0.49–1.37)	0.45	0.96	(0.39–2.29)	0.87
**Parity**	**Primipara vs. Multipara**	1.28	(0.77–2.14)	0.33	1.99	(0.99–4.06)	0.06
**IVF pregnancy**	**Yes vs. No**	2.17	(0.86–5.59)	0.10	1.89	(0.62–5.94)	0.26
**Repeat cesarean section**	**Yes vs. No**	1.10	(0.51–2.30)	0.81	1.24	(0.42–3.52)	0.69
**Tocolytic agent use**	**Yes vs. No**	1.16	(0.69–1.94)	0.58	1.11	(0.56–2.21)	0.77
**Warning bleeding**	**Yes vs. No**	1.12	(0.65–1.91)	0.68	0.67	(0.28–1.54)	0.35
**Cesarean section mode**	**Emergency vs. Elective**	1.36	(0.78–2.39)	0.28	1.70	(0.66–4.58)	0.27
**Myoma-related complication**	**Yes vs. No**	0.70	(0.15–2.60)	0.61	0.65	(0.11–3.03)	0.60
**Previa classification**	**Major previa vs. Minor previa**	1.32	(0.79–2.20)	0.29	1.05	(0.53–2.08)	0.89
**Main placenta location**	**Anterior wall vs. Posterior wall**	1.53	(0.76–3.03)	0.23	1.40	(0.59–3.31)	0.44
**Uterine incision type**	**Transverse vs. Classical**	0.89	(0.31–2.74)	0.84	3.67	(0.65–31.61)	0.14
**Placental adhesion**	**Yes vs. No**	4.70	(1.94–12.60)	0.0005	12.68	(2.85–92.13)	0.0004
**Hemostatic procedures needed**	**Yes vs. No**	3.31	(1.86–5.34)	<0.0001	3.31	(1.79–6.25)	0.0001
**Birth weight**	**≥−0.33 SD vs. <−0.33 SD**	5.08	(2.86–9.37)	<0.0001	5.88	(3.04–12.00)	<0.0001

OR, odds ratio; CI, confidence interval; IVF, in vitro fertilization; SD, standard deviation

## Discussion

Based on the results of the multivariate analysis adjusted for conventional factors, our study findings suggested that predicting hemorrhage amount during cesarean section for placental previa by using the SD value of the birth weight had the appropriate AUC (0.71) and high sensitivity (81.3%). It was well-known that massive hemorrhage in placenta previa results in high mortality for mothers. This fact has induced several recommendations for preparing for treatment in case of massive hemorrhage. Thus, predicting hemorrhage with high sensitivity for placental previa is significantly useful in clinical settings.

A well-known mechanism of bleeding in placenta previa is poor contraction in the lower uterine segment after delivery [18]. We hypothesized that massive bleeding more frequently occurred in cases of increasing placental blood flow in addition to poor contraction in placenta previa. However, accurately evaluating placental blood flow was difficult. Because placental blood flow is associated with fetal growth [19,20], we substituted fetal weight as a marker of placental blood flow. Establishing this connection required standardization of the gestational ages of all included cases. However, since this study included cases with various gestational ages, the differences required normalization. Thus, we calculated SD values according to the Japanese standard growth curve at each gestational age to achieve a more accurate assessment of placental blood flow among groups.

On the other hand, no association was found between birth weight and hemorrhage in women who underwent cesarean section [21]. This is because a low proportion of patients without placenta previa are at potential risk of bleeding, such as those with poor contraction in the lower uterine segment. Therefore, this association between birth weight and hemorrhage might be unique to cases with placenta previa.

There have been considerable arguments about the correlation between placenta previa and low birth weight in several countries [22–25]. In our study, the mean overall birth weight was considered low birth weight because it was −0.11 SD of the standard birth weight. These differences might have arisen from race and country. However, our analysis demonstrated that the SD values of birth weight were strongly associated with massive bleeding. We believe this concept would be accepted if the appropriate SD values of fetal weight are calculated and recommend the use of this concept combined with other predictors to screen for intraoperative hemorrhage during cesarean section. The use of an additional 36-week fetal weight scan, which is not currently universal, might be an effective antenatal care protocol for patients with placenta previa.

The need for hemostatic procedures was identified as a risk factor in this study because such procedures were performed for patients with increased bleeding. Placenta accreta has already been established as strong risk factor of intraoperative hemorrhage [8,14].

This study has several limitations such as the fact that it was conducted in a single institution and included only Asian women. Our findings will need to be evaluated in several other countries to verify their validity. In addition, birth weight was measured after the cesarean section in the present study. Obviously, a preoperative prediction would be most useful; therefore, further studies are required to evaluate whether fetal weight estimated by ultrasonography can predict hemorrhage during cesarean section in patients with placental previa. In conclusion, fetal weight may be a useful marker for predicting massive hemorrhage during cesarean section in patients with placenta previa.

## Supporting Information

S1 File This is the data set used for this study. (PDF)
