# Supplementary material for: Relation between Birth Weight and Intraoperative Hemorrhage during Cesarean Section in Pregnancy with Placenta Previa
Source: PLoS One. 2016 Nov 30;11(11):e0167332. doi: 10.1371/journal.pone.0167332 (PMC5130260; doi:10.1371/journal.pone.0167332)
Supplement: S1 File — (PDF) [file pone.0167332.s001.pdf]

case	age	gestational age	Birth weight (g)	SD of birth weight (SD)	parity	IVF pregnancy	peat cesarean secti	cesarean section mode	previa classification	main placenta location	uterine incision type	placental adhesion	warning bleeding	hemostatic procedures	tocolytic agent use	Intraoperative hemorrhage (ml)	myoma
1	38	39w1d	2680	−0.93141913	primipara	no	no	elective	major	posterior	transverse	no	no	yse	no	1526	no
2	29	38w4d	2680	−0.717271782	primipara	yes	no	elective	minor	posterior	transverse	no	no	no	no	904	no
3	29	38w1d	3060	0.542102575	multipara	no	no	emergency	minor	posterior	transverse	no	yes	yse	no	2241	no
4	30	38w1d	3365	1.312722053	primipara	no	no	elective	minor	posterior	transverse	no	no	yse	no	829	no
5	34	38w0d	2730	−0.346461772	primipara	no	no	elective	minor	posterior	transverse	no	no	no	yes	1585	no
6	36	38w0d	2978	2.13970617	multipara	no	yes	elective	minor	posterior	transverse	no	no	no	no	1348	no
7	37	38w0d	2945	0.306518674	primipara	no	no	emergency	minor	posterior	transverse	no	no	yse	no	1233	no
8	31	37w6d	2990	0.463864284	primipara	no	no	elective	minor	posterior	transverse	no	no	no	no	1538	yes
9	35	37w6d	2975	0.440161676	multipara	no	no	elective	minor	posterior	transverse	no	no	yse	no	1727	no
10	31	37w6d	2858	0.001312041	primipara	no	no	elective	minor	posterior	transverse	no	no	no	no	1555	no
11	35	37w6d	3510	1.530334023	multipara	no	no	elective	minor	posterior	transverse	no	yes	no	no	1128	no
12	35	37w6d	3020	0.577393747	multipara	no	no	elective	minor	posterior	transverse	no	no	no	no	1221	no
13	33	37w6d	2965	0.4324567	multipara	no	yes	elective	minor	posterior	transverse	no	no	no	no	896	no
14	37	37w6d	2675	−0.429042179	multipara	no	no	elective	minor	posterior	transverse	no	no	no	yes	939	yes
15	33	37w6d	2636	−0.555876398	primipara	no	no	elective	minor	posterior	transverse	no	no	yse	no	1076	no
16	42	37w6d	2631	2.431613381	primipara	no	no	elective	minor	posterior	transverse	no	no	no	no	525	no
17	30	37w6d	2595	−0.671461694	multipara	no	no	elective	minor	posterior	transverse	no	no	no	no	831	no
18	27	37w6d	2290	−1.608517648	multipara	no	no	elective	major	posterior	transverse	no	no	yse	no	1153	no
19	35	37w6d	2005	−2.484127311	multipara	no	yes	elective	minor	anterior	transverse	no	no	no	yes	1418	no
20	32	37w5d	3470	1.444280274	primipara	no	no	elective	minor	posterior	transverse	no	no	no	no	1523	no
21	34	37w5d	3250	1.245918412	primipara	yes	no	elective	minor	posterior	transverse	no	no	no	no	2640	no
22	28	37w5d	3065	0.742380374	multipara	no	no	elective	minor	posterior	transverse	no	no	yse	no	1891	no
23	29	37w5d	2995	0.574585579	primipara	no	no	elective	minor	posterior	transverse	no	no	yse	no	1810	no
24	37	37w5d	2964	0.462708502	multipara	no	yes	elective	major	anterior	transverse	no	no	yse	no	2838	no
25	31	37w5d	2875	0.171488711	multipara	no	no	elective	minor	posterior	transverse	no	no	yse	yes	1572	no
26	36	37w5d	2738	−0.176780676	multipara	no	no	elective	minor	posterior	transverse	no	no	no	no	2023	no
27	32	37w5d	3820	1.737513118	primipara	no	no	elective	major	posterior	transverse	no	no	no	no	853	no
28	41	37w5d	3110	0.901442847	primipara	yes	no	elective	minor	posterior	transverse	no	no	no	no	1268	no
29	38	37w5d	2970	0.46529471	primipara	no	no	elective	minor	anterior	transverse	no	no	yse	no	1341	no
30	33	37w5d	2895	0.268651679	multipara	no	yes	elective	minor	posterior	transverse	no	no	no	no	1009	no
31	27	37w5d	2754	1.809487233	primipara	no	no	elective	minor	posterior	transverse	no	no	no	no	798	no
32	33	37w5d	2730	−0.20916117	multipara	no	no	elective	minor	posterior	transverse	no	no	no	no	1114	no
33	29	37w5d	2455	−1.04085021	multipara	no	no	elective	minor	posterior	transverse	no	no	no	no	1016	no
34	33	37w5d	2414	−1.167908556	multipara	no	no	elective	minor	posterior	transverse	no	no	no	no	1004	no
35	37	37w4d	2760	−0.079639194	multipara	no	no	elective	major	posterior	transverse	yes	yes	yse	no	6223	no
36	32	37w4d	3276	1.334214846	primipara	no	no	elective	major	posterior	transverse	no	no	yse	no	1786	no
37	33	37w4d	3155	1.019530311	primipara	no	no	elective	minor	posterior	transverse	no	no	yse	no	1644	no
38	43	37w4d	2916	0.373687724	primipara	yes	no	elective	minor	posterior	transverse	no	no	yse	no	1533	no
39	35	37w4d	2910	0.342769215	primipara	no	no	elective	major	posterior	transverse	no	yes	yse	yes	2542	no
40	35	37w4d	2598	−0.540673538	multipara	no	no	elective	major	anterior	transverse	no	no	yse	no	2284	no
41	36	37w4d	2930	0.443952354	multipara	no	no	elective	major	posterior	classical	yes	no	no	no	544	no
42	34	37w4d	3230	1.300307557	primipara	no	no	elective	minor	posterior	transverse	no	no	yse	no	1244	no
43	42	37w4d	2750	−0.100650733	primipara	yes	no	elective	minor	posterior	transverse	no	no	no	no	403	no
44	38	37w4d	2736	1.759471073	multipara	no	no	elective	minor	posterior	transverse	no	yes	no	yes	820	no
45	33	37w4d	2562	−0.643904259	multipara	no	no	elective	major	posterior	transverse	no	no	no	no	1125	no
46	40	37w4d	2550	−0.681416556	multipara	no	yes	elective	major	posterior	transverse	no	no	no	no	570	no
47	37	37w4d	2490	−0.868978041	multipara	no	no	elective	major	posterior	transverse	no	no	no	no	690	no
48	34	37w4d	2380	−1.212840764	multipara	no	no	elective	major	posterior	transverse	no	no	yse	no	714	no
49	30	37w4d	2288	−1.500435041	primipara	yes	no	elective	minor	posterior	transverse	no	no	no	yes	1150	no
50	38	37w4d	2220	−1.713004724	multipara	no	yes	elective	major	posterior	transverse	no	yes	yse	yes	1348	no
51	28	37w3d	3040	0.849530047	multipara	no	no	elective	major	posterior	transverse	yes	no	yse	yes	3258	no
52	36	37w3d	3516	1.588077412	multipara	no	no	elective	major	posterior	transverse	no	no	yse	no	1676	no
53	36	37w3d	3405	1.444125643	primipara	no	no	elective	major	anterior	transverse	no	no	yse	yes	2340	no
54	35	37w3d	3168	1.203661546	multipara	no	yes	elective	minor	posterior	transverse	no	yes	yse	yes	2851	no
55	38	37w3d	3140	1.037487853	primipara	no	no	elective	minor	posterior	transverse	no	no	no	yes	1533	no
56	28	37w3d	2845	0.265192955	primipara	no	no	elective	major	posterior	transverse	no	yes	yse	no	1698	no
57	42	37w3d	3084	0.949161589	multipara	no	no	elective	major	posterior	classical	no	no	no	no	1225	no
58	36	37w3d	3140	1.069325544	multipara	yes	no	elective	minor	posterior	transverse	no	no	no	no	598	no
59	34	37w3d	2985	0.615572347	multipara	no	no	elective	minor	posterior	transverse	no	no	no	no	336	no
60	26	37w3d	2892	0.42158522	primipara	no	no	elective	minor	posterior	transverse	no	no	no	yes	1318	no
61	37	37w3d	2790	0.057400402	multipara	no	no	emergency	minor	posterior	transverse	no	no	yse	yes	957	no
62	39	37w3d	2740	−0.056211606	multipara	no	no	elective	major	posterior	transverse	no	yes	no	no	304	no
63	26	37w3d	2615	−0.425676525	primipara	no	no	elective	major	posterior	transverse	no	no	no	no	724	yes
64	31	37w3d	2555	−0.598846366	primipara	no	no	elective	major	posterior	transverse	no	no	no	no	1156	no
65	25	37w3d	2550	−0.614613587	multipara	no	yes	elective	minor	posterior	transverse	no	yes	no	no	265	no
66	34	37w3d	2540	−0.646148027	multipara	no	no	elective	minor	posterior	transverse	no	no	yse	no	1079	no
67	34	37w3d	2500	−0.77228579	multipara	no	no	elective	minor	posterior	transverse	no	no	no	no	532	no
68	30	37w3d	2400	−1.087630197	primipara	no	no	elective	minor	posterior	transverse	no	no	yse	no	748	no
69	31	37w3d	2310	−1.371440162	multipara	no	yes	elective	major	posterior	classical	no	no	no	no	879	no
70	30	37w3d	2260	−1.529112366	primipara	no	no	elective	minor	posterior	transverse	no	yes	no	yes	303	no
71	36	37w3d	2238	−1.598488135	multipara	no	no	elective	major	posterior	transverse	no	no	no	no	967	no
72	37	37w3d	2106	−2.014742752	multipara	no	no	elective	major	posterior	transverse	no	no	no	yes	1004	no
73	32	37w2d	2667	−0.182406337	multipara	no	yes	elective	major	posterior	transverse	yes	no	yse	no	4936	no
74	41	37w2d	2955	0.615328999	primipara	no	no	elective	minor	posterior	transverse	no	no	no	no	1653	no
75	41	37w2d	2900	0.496017856	primipara	no	no	elective	major	posterior	transverse	no	no	yse	yes	2020	no
76	27	37w2d	2860	0.33972789	primipara	no	no	elective	minor	posterior	transverse	no	no	yse	no	1616	no
77	30	37w2d	2805	0.198833055	primipara	no	no	elective	major	posterior	transverse	no	no	no	no	2168	no
78	36	37w2d	2690	−0.124971886	primipara	no	no	elective	minor	anterior	transverse	no	no	no	no	1577	no
79	41	37w2d	2634	−0.286874356	primipara	no	no	elective	minor	posterior	transverse	no	yes	no	yes	1821	no
80	37	37w2d	2530	−0.609650647	multipara	no	no	elective	major	posterior	transverse	no	no	no	no	1768	no
81	31	37w2d	2350	−1.18227555	primipara	no	no	elective	major	posterior	transverse	no	no	no	no	1786	no
82	40	37w2d	3100	0.999099698	multipara	no	no	elective	minor	posterior	transverse	no	no	yse	no	829	no
83	37	37w2d	3020	0.872482614	multipara	no	yes	elective	major	anterior	transverse	no	no	no	no	1474	no
84	29	37w2d	3005	0.8093579	multipara	no	no	elective	major	posterior	transverse	no	no	yse	no	834	no
85	41	37w2d	2945	0.609215308	primipara	no	no	elective	minor	posterior	transverse	no	no	yse	yes	1419	no
86	40	37w2d	2940	0.602345422	primipara	no	no	elective	minor	posterior	transverse	no	no	no	yes	880	no
87	31	37w2d	2880	0.44193106	primipara	no	no	elective	major	posterior	transverse	no	yes	no	yes	306	no
88	38	37w2d	2690	−0.115113409	multipara	yes	no	elective	minor	posterior	transverse	no	no	no	no	489	no
89	40	37w2d	2594	−0.418775679	primipara	no	no	elective	minor	posterior	transverse	no	no	no	no	1073	no
90	38	37w2d	2590	−0.427435832	primipara	no	no	elective	major	anterior	transverse	no	no	no	no	1212	no
91	34	37w2d	2586	−0.449964593	multipara	no	no	elective	minor	posterior	transverse	no	no	no	yes	1087	no
92	38	37w2d	2525	−0.625556894	multipara	no	no	elective	minor	posterior	transverse	no	no	no	no	909	no
93	36	37w2d	2506	−0.686000634	multipara	no	no	elective	minor	posterior	transverse	no	no	no	yes	678	no
94	37	37w2d	2445	−0.880056851	primipara	no	no	elective	minor	posterior	transverse	no	no	no	no	472	no
95	36	37w2d	2400	−1.023213077	primipara	no	no	elective	minor	posterior	transverse	no	no	no	no	646	no
96	31	37w2d	2374	−1.105925563	primipara	no	no	elective	minor	posterior	transverse	no	no	yse	no	520	no
97	33	37w2d	2344	−1.201363046	multipara	no	no	elective	major	posterior	transverse	no	yes	yse	no	992	no
98	26	37w1d	2415	−0.908915746	multipara	no	yes	elective	major	posterior	transverse	yes	no	yse	no	2400	no
99	30	37w1d	3262	1.422842093	multipara	no	yes	elective	minor	posterior	transverse	no	no	no	no	1510	no
100	35	37w1d	3138	1.209828417	primipara	no	no	elective	minor	posterior	transverse	no	no	no	yes	2245	yes
101	40	37w1d	3040	0.988984329	primipara	yes	no	elective	major	posterior	transverse	no	no	no	no	1728	no
102	34	37w1d	2990	0.863748182	primipara	no	no	elective	minor	posterior	transverse	no	no	yse	no	2495	no
103	29	37w1d	2890	0.567411654	primipara	no	no	elective	minor	posterior	transverse	no	no	yse	no	1952	no
104	35	37w1d	2880	0.531872493	multipara	yes	no	elective	minor	anterior	transverse	no	no	yse	no	1596	no
105	35	37w1d	2789	0.241026247	primipara	no	no	elective	major	posterior	transverse	no	yes	yse	yes	2561	no
106	36	37w1d	2595	−0.359256738	primipara	no	no	elective	major	posterior	transverse	no	no	no	no	1712	no
107	33	37w1d	2525	−0.5608583	primipara	no	no	elective	minor	anterior	transverse	no	no	no	no	2202	no
108	39	37w1d	2326	−1.19455665	primipara	no	no	elective	major	posterior	transverse	no	no	no	yes	1562	no
109	31	37w1d	2970	0.759997535	primipara	no	no	elective	minor	posterior	transverse	no	no	yse	no	1091	no
110	35	37w1d	2935	0.632601165	multipara	no	no	elective	minor	posterior	transverse	no	no	no	no	572	no
111	31	37w1d	2805	0.323987953	multipara	no	no	emergency	minor	posterior	transverse	no	no	no	no	565	no
112	37	37w1d	2672	−0.11425396	multipara	no	no	elective	major	posterior	transverse	no	no	no	no	1159	no
113	37	37w1d	2638	−0.211676895	multipara	no	no	elective	major	posterior	transverse	no	no	yse	no	1381	no
114	42	37w1d	2610	−0.294708653	multipara	no	no	elective	minor	posterior	transverse	no	no	no	no	623	no
115	39	37w1d	2558	−0.500197809	primipara	no	no	elective	minor	posterior	transverse	no	no	no	no	1400	no
116	30	37w1d	2515	−0.587970884	primipara	no	no	elective	minor	posterior	transverse	no	no	yse	no	764	yes
117	31	37w1d	2472	−0.72597715	primipara	no	no	elective	major	posterior	transverse	no	no	no	yes	1208	no
118	34	37w1d	2456	−0.777328352	multipara	no	no	elective	minor	posterior	transverse	no	no	no	no	1248	no
119	41	37w1d	2445	−0.812632287	primipara	no	no	elective	major	anterior	classical	yes	yes	yse	no	720	no
120	35	37w1d	2430	−0.860774016	multipara	no	no	elective	minor	posterior	transverse	no	no	no	no	1119	no
121	34	37w1d	2388	−0.99635016	multipara	no	no	elective	major	posterior	transverse	no	no	no	yes	899	no
122	33	37w1d	2312	−1.23948893	multipara	no	no	elective	minor	posterior	transverse	no	no	no	yes	847	no
123	30	37w1d	2232	−1.496244842	multipara	no	yes	elective	major	anterior	transverse	no	yes	no	yes	1352	no
124	41	37w1d	2118	−1.8621219	primipara	no	no	elective	minor	posterior	transverse	no	no	no	yes	670	no
125	33	37w0d	2670	−0.054991	multipara	no	yes	elective	major	posterior	transverse	yes	yes	yse	no	1943	no
126	33	37w0d	3080	1.09698964	primipara	yes	no	elective	minor	posterior	transverse	no	no	yse	no	2196	no
127	33	37w0d	3048	1.015582141	primipara	no	no	elective	major	anterior	transverse	no	no	yse	yes	1895	no
128	33	37w0d	2736	0.142212989	primipara	no	no	elective	major	posterior	transverse	no	no	yse	no	2178	no
129	36	37w0d	2680	−0.015459214	primipara	no	no	elective	major	posterior	transverse	no	no	no	no	1848	no
130	27	37w0d	2636	−0.128209935	multipara	no	no	elective	minor	posterior	transverse	no	yes	yse	yes	2039	no
131	40	37w0d	2635	−0.17381947	multipara	no	no	elective	minor	posterior	transverse	no	yes	yse	yes	1595	no
132	35	37w0d	2586	−0.301622004	primipara	yes	no	elective	major	posterior	transverse	no	no	yse	no	1514	yes
133	29	37w0d	3080	1.19956	multipara	no	yes	elective	minor	anterior	transverse	no	no	no	yes	643	no
134	39	37w0d	2995	0.946355882	multipara	no	no	elective	minor	posterior	transverse	no	no	no	no	1258	no
135	31	37w0d	2790	0.331938418	primipara	no	no	elective	minor	posterior	transverse	no	no	yse	no	1198	no
136	36	37w0d	2676	−0.024951359	primipara	no	no	elective	major	posterior	transverse	no	no	no	yes	698	no
137	31	37w0d	2675	−0.03538864	primipara	no	no	elective	major	posterior	transverse	no	no	yse	no	1356	no
138	28	37w0d	2650	−0.100650733	primipara	no	no	elective	minor	posterior	transverse	no	no	no	no	978	no
139	31	37w0d	2620	−0.188629602	primipara	no	no	elective	minor	posterior	transverse	no	no	no	no	798	no
140	27	37w0d	2610	−0.22060104	primipara	no	no	elective	major	posterior	transverse	no	no	no	yes	1030	no
141	31	37w0d	2578	−0.331214995	multipara	no	no	elective	minor	posterior	transverse	no	no	yse	no	1258	no
142	34	37w0d	2520	−0.505786746	primipara	no	no	elective	major	posterior	transverse	no	no	no	no	1295	no
143	25	37w0d	2500	−0.565346606	primipara	no	no	elective	major	posterior	transverse	no	no	yse	no	1375	no
144	36	37w0d	2475	−0.646297841	multipara	no	yes	elective	major	posterior	transverse	no	no	yse	no	1067	no
145	27	37w0d	2448	−0.733725175	primipara	no	no	elective	minor	anterior	transverse	no	no	no	no	543	no
146	35	37w0d	2386	−0.934484239	primipara	no	no	elective	major	posterior	transverse	no	no	no	no	678	no
147	34	37w0d	2350	−1.051054018	primipara	no	no	elective	minor	posterior	transverse	no	no	yse	no	1215	yes
148	31	37w0d	2350	−1.051054018	primipara	no	no	elective	major	posterior	transverse	no	no	no	no	767	no
149	33	37w0d	2332	−1.109338907	primipara	yes	no	elective	minor	posterior	transverse	no	no	no	yes	975	no
150	29	36w6d	2410	−0.786812425	primipara	no	no	elective	major	posterior	transverse	yes	no	yse	no	1588	no
151	31	36w6d	2634	−0.09997593	primipara	no	no	elective	minor	anterior	classical	no	yes	no	yes	1573	no
152	29	36w6d	2595	−0.193208704	primipara	no	no	elective	minor	posterior	transverse	no	no	no	no	1507	no
153	34	36w6d	2332	−1.0416431	primipara	yes	no	elective	minor	posterior	transverse	no	no	yse	yes	2045	no
154	40	36w6d	1945	−2.305995294	multipara	no	no	elective	major	posterior	transverse	no	no	yse	no	2070	no
155	35	36w6d	3018	3.189262989	primipara	no	no	elective	minor	posterior	transverse	no	no	no	yes	850	no
156	42	36w6d	2750	0.280801981	primipara	no	no	elective	minor	posterior	transverse	no	no	no	no	978	no
157	33	36w6d	2698	0.136557048	primipara	no	no	elective	major	anterior	transverse	no	no	no	yes	913	yes
158	32	36w6d	2640	−0.084087451	primipara	no	no	emergency	major	posterior	transverse	no	yes	no	yes	280	no
159	36	36w6d	2520	−0.431500677	primipara	no	no	elective	major	posterior	transverse	no	yes	no	no	1230	no
160	26	36w6d	2400	−0.819483024	primipara	no	no	elective	major	posterior	transverse	no	no	yse	no	1295	no
161	37	36w6d	2400	−0.819483024	primipara	no	no	elective	minor	posterior	transverse	no	no	no	yes	1100	no
162	37	36w6d	2117	−1.744060985	primipara	no	no	elective	major	posterior	transverse	no	no	no	no	1016	no
163	38	36w5d	2505	−0.409639722	multipara	no	yes	emergency	major	posterior	transverse	yes	yes	yse	no	1736	no
164	36	36w5d	3154	1.390941421	primipara	no	no	emergency	minor	anterior	transverse	no	no	no	no	2359	no
165	27	36w5d	3065	1.245918412	primipara	no	no	elective	major	posterior	transverse	no	no	no	yes	1501	no
166	42	36w5d	2930	0.908532642	multipara	no	no	elective	major	posterior	transverse	no	no	no	no	1834	no
167	29	36w5d	2914	0.872328237	multipara	no	no	emergency	minor	posterior	transverse	no	no	no	yes	2589	no
168	36	36w5d	2670	0.017502288	primipara	no	no	elective	minor	posterior	transverse	no	no	yse	no	2192	no
169	35	36w5d	2335	−0.962310948	primipara	yes	no	elective	minor	posterior	transverse	no	no	yse	no	1572	no
170	33	36w5d	2400	−0.748039197	multipara	no	yes	elective	major	anterior	classical	yes	yes	no	yes	1059	no
171	34	36w5d	2935	0.930574005	multipara	no	yes	elective	major	posterior	transverse	no	yes	no	yes	1364	no
172	36	36w5d	2766	0.345217041	multipara	no	no	elective	major	posterior	transverse	no	yes	no	no	891	no
173	31	36w5d	2692	2.081581089	multipara	no	yes	elective	major	posterior	transverse	no	yes	no	yes	770	no
174	35	36w5d	2592	−0.125960211	multipara	no	no	elective	minor	posterior	transverse	no	no	no	yes	727	no
175	29	36w5d	2560	−0.251432571	primipara	no	no	emergency	minor	anterior	transverse	no	no	no	yes	984	no
176	38	36w5d	2516	−0.401907907	primipara	no	no	elective	minor	anterior	transverse	no	no	no	yes	1427	no
177	31	36w5d	2505	−0.406050681	multipara	no	yes	elective	major	posterior	transverse	no	no	no	no	1249	no
178	19	36w5d	2402	−0.74144622	primipara	no	no	emergency	minor	posterior	transverse	no	yes	no	yes	1083	no
179	22	36w5d	2348	−0.919456598	primipara	no	no	elective	minor	posterior	transverse	no	no	no	no	450	no
180	41	36w5d	2159	−1.542492921	primipara	no	no	elective	major	posterior	transverse	no	no	yse	no	1355	yes
181	24	36w4d	2926	0.951814386	multipara	no	yes	elective	major	anterior	classical	yes	no	yse	yes	3154	no
182	41	36w4d	2565	−0.16383773	multipara	yes	no	emergency	major	posterior	transverse	yes	yes	no	no	2325	no
183	42	36w4d	2876	0.839850615	multipara	no	no	emergency	major	posterior	transverse	no	yes	yse	no	2106	no
184	37	36w4d	2380	−0.741333772	multipara	no	no	elective	major	posterior	transverse	no	no	yse	no	757	no
185	30	36w4d	2210	−1.30681218	primipara	no	no	emergency	minor	posterior	transverse	no	yes	yse	yes	1299	no
186	29	36w4d	2006	−1.98538627	primipara	no	no	elective	major	posterior	transverse	no	no	yse	no	1267	no
187	31	36w3d	2680	0.322702762	primipara	no	no	elective	major	posterior	transverse	yes	no	yse	no	2774	no
188	29	36w3d	2670	0.260723082	multipara	no	yes	elective	major	anterior	classical	yes	yes	no	yes	1674	no
189	38	36w3d	2705	0.315652413	multipara	no	no	emergency	major	posterior	transverse	no	yes	no	no	714	no
190	34	36w3d	2428	−0.531367368	multipara	no	no	elective	major	posterior	transverse	no	yes	yse	yes	1176	no
191	34	36w2d	2724	0.503786665	primipara	yes	no	elective	major	posterior	transverse	yes	no	no	yes	2289	no
192	30	36w2d	2355	−0.675353448	primipara	no	no	elective	major	posterior	transverse	yes	yes	yse	no	1295	no
193	33	36w2d	2965	1.209104472	primipara	no	no	emergency	major	posterior	transverse	no	yes	yse	yes	1473	no
194	35	36w2d	2744	0.583477861	multipara	no	no	elective	major	posterior	transverse	no	yes	no	yes	531	no
195	23	36w2d	2605	0.142012693	primipara	no	no	elective	major	posterior	transverse	no	yes	no	yes	1259	no
196	42	36w2d	2418	2.170955612	primipara	no	no	elective	minor	posterior	transverse	no	no	no	yes	1473	no
197	21	36w1d	3165	1.545939368	multipara	no	no	emergency	major	anterior	classical	yes	yes	no	yes	4700	no
198	31	36w1d	2255	−0.93993109	multipara	no	yes	emergency	minor	posterior	transverse	no	yes	yse	yes	1779	no
199	41	36w1d	2762	0.555126565	primipara	yes	no	elective	major	posterior	transverse	no	no	no	yes	437	yes
200	32	36w1d	2580	0.1374686	multipara	no	no	emergency	minor	posterior	transverse	no	yes	no	no	472	no
201	41	36w1d	2246	−0.970698101	multipara	yes	no	emergency	minor	posterior	transverse	no	yes	no	yes	724	no
202	33	36w1d	2020	−1.743291942	multipara	no	no	emergency	major	posterior	transverse	no	yes	yse	yes	637	no
203	37	36w0d	2740	0.694661632	multipara	no	no	elective	major	posterior	transverse	yes	no	yse	yes	4627	no
204	36	36w0d	2680	0.53653266	multipara	no	no	emergency	major	posterior	transverse	no	yes	no	yes	1655	no
205	29	36w0d	2420	−0.312778752	primipara	no	no	emergency	major	anterior	transverse	no	yes	no	yes	1270	no
206	35	36w0d	2405	−0.365646533	multipara	no	no	elective	major	posterior	transverse	no	yes	yse	yes	1097	no
207	32	36w0d	2080	−1.467779365	multipara	no	yes	elective	major	anterior	classical	yes	yes	no	no	672	no
208	22	36w0d	2030	−1.640289764	primipara	no	no	emergency	major	posterior	transverse	no	yes	no	yes	590	no
209	30	35w6d	2550	0.171099875	primipara	no	no	emergency	major	anterior	transverse	no	yes	no	yes	1665	no
210	32	35w6d	2420	−0.249989993	primipara	no	no	emergency	major	posterior	transverse	no	no	yse	yes	2250	no
211	31	35w6d	2540	0.154100836	multipara	no	yes	emergency	major	posterior	transverse	no	yes	no	yes	1188	no
212	41	35w5d	2695	0.71056788	multipara	no	no	emergency	major	posterior	transverse	no	yes	yse	yes	2666	no
213	32	35w5d	2334	−0.433129043	multipara	no	no	emergency	minor	anterior	transverse	no	yes	yse	no	1878	no
214	38	35w5d	2826	1.074849821	primipara	no	no	emergency	major	anterior	transverse	no	yes	no	no	1196	no
215	33	35w5d	2084	−1.307776801	multipara	no	no	emergency	major	posterior	transverse	no	yes	no	yes	1132	no
216	30	35w4d	2470	0.004550091	primipara	no	no	emergency	minor	posterior	transverse	no	yes	yse	yes	1913	no
217	22	35w4d	2330	−0.401907907	primipara	no	no	emergency	minor	posterior	transverse	no	yes	no	yes	769	no
218	32	35w3d	2838	1.312722053	multipara	no	no	emergency	major	anterior	transverse	no	yes	no	yes	1713	no
219	28	35w3d	1690	−2.56578492	primipara	no	no	emergency	major	posterior	transverse	no	yes	yse	yes	510	no
220	18	35w2d	1410	−3.512416057	primipara	no	no	emergency	major	posterior	classical	yes	yes	no	yes	1058	no
221	25	35w2d	2298	−0.322953595	multipara	no	no	emergency	major	anterior	transverse	no	no	no	yes	699	no
222	37	35w1d	2430	0.206202929	multipara	no	no	emergency	major	posterior	transverse	no	yes	yse	yes	2719	no
223	34	35w1d	1618	−2.695475086	multipara	no	no	emergency	major	posterior	transverse	no	no	no	no	542	no
224	35	35w0d	2488	0.506472849	multipara	no	no	emergency	major	posterior	transverse	no	yes	yse	no	1013	no
225	23	35w0d	2061	−0.99557083	multipara	no	no	emergency	major	posterior	transverse	no	yes	yse	no	1431	no
226	29	34w4d	2190	−0.278800703	multipara	no	no	emergency	major	posterior	transverse	no	yes	yse	yes	1457	no
227	32	34w3d	1864	−1.405053546	primipara	no	no	emergency	major	posterior	classical	yes	yes	no	no	3408	no
228	38	34w3d	2316	0.291418037	primipara	yes	no	emergency	major	posterior	transverse	no	yes	yse	yes	1801	no
229	28	34w3d	2346	0.340085013	multipara	no	no	emergency	minor	anterior	transverse	no	yes	yse	yes	1159	no
230	35	34w2d	1890	−1.220746006	primipara	no	no	emergency	minor	posterior	transverse	no	no	yse	yes	663	no
231	39	34w2d	2278	0.21453547	multipara	no	yes	elective	major	anterior	classical	yes	yes	no	no	501	no
232	34	34w0d	2355	0.634272014	multipara	no	no	emergency	minor	posterior	transverse	no	no	no	yes	1632	no
233	36	34w0d	2018	−0.545496795	multipara	no	no	emergency	major	posterior	transverse	no	yes	yse	no	1927	no
234	36	33w6d	1878	−1.003765536	primipara	no	no	emergency	major	anterior	classical	no	no	yse	no	800	no
235	43	33w5d	1681	−1.78937383	multipara	no	yes	emergency	minor	posterior	transverse	no	no	yse	yes	1190	no
236	32	33w3d	2068	−0.014878206	multipara	no	no	emergency	major	posterior	classical	yes	yes	no	yes	2336	no
237	34	33w3d	2302	0.936517209	multipara	no	no	emergency	major	posterior	transverse	no	yes	no	yes	1013	no
238	23	33w3d	2138	0.326973512	multipara	no	no	emergency	major	posterior	transverse	no	yes	yse	no	1238	no
239	28	33w3d	1748	−1.258332807	multipara	no	no	emergency	major	posterior	transverse	no	yes	no	no	1100	no
240	33	33w1d	2196	0.642332952	multipara	no	no	emergency	major	anterior	transverse	no	yes	yse	yes	2760	no
241	38	33w0d	2104	0.458475587	multipara	no	yes	emergency	minor	posterior	transverse	no	no	no	no	2750	no
242	29	33w0d	2078	2.104618579	multipara	no	no	emergency	minor	anterior	transverse	no	no	no	yes	1611	no
243	37	33w0d	1692	−1.215757072	primipara	no	no	emergency	minor	posterior	transverse	no	no	no	no	1518	no
244	31	32w6d	2364	1.411515604	multipara	no	yes	emergency	major	posterior	transverse	no	yes	yse	yes	3103	no
245	32	32w5d	1430	−2.159843089	multipara	no	no	emergency	minor	posterior	transverse	no	no	no	no	1000	no
246	33	32w3d	1574	−1.348567603	primipara	no	no	emergency	minor	posterior	transverse	no	yes	no	no	614	no
247	36	32w1d	2022	0.789777284	multipara	no	no	emergency	major	anterior	transverse	no	yes	yse	yes	1097	no
248	26	31w6d	1516	−1.21985893	multipara	no	no	emergency	major	posterior	transverse	no	yes	no	yes	634	no
249	40	31w4d	1918	0.807141973	multipara	no	yes	emergency	major	posterior	transverse	yes	yes	no	yes	1649	no
250	29	31w3d	1654	−0.283073265	multipara	no	no	emergency	major	anterior	transverse	no	yes	no	no	1500	no
251	30	31w3d	1616	−0.46194921	primipara	no	no	emergency	major	posterior	transverse	no	yes	no	yes	988	no
252	33	31w1d	1348	−1.51766299	primipara	no	no	emergency	major	posterior	transverse	no	no	yse	no	2111	no
253	40	31w1d	1556	−0.500585618	primipara	no	no	emergency	major	posterior	transverse	no	yes	yse	no	1251	no
254	40	31w1d	1114	−2.662311319	primipara	no	no	emergency	minor	posterior	transverse	no	yes	no	yes	742	no
255	35	30w3d	1706	0.757134582	multipara	no	no	emergency	major	posterior	transverse	no	yes	yse	yes	1697	no
256	31	30w1d	1672	0.884571986	multipara	no	no	emergency	major	anterior	transverse	no	no	yse	no	1378	no
